# Ocular myasthenia gravis management

**DOI:** 10.3389/fneur.2025.1678151

**Published:** 2025-11-03

**Authors:** Mark J. Kupersmith, Gil I. Wolfe, Henry Kaminski

**Affiliations:** ^1^Department of Ophthalmology, Icahn School of Medicine at Mount Sinai, New York Eye and Ear Infirmary and Union Square Eye Care, New York, NY, United States; ^2^Department of Neurology, Jacobs School of Medicine and Biomedical Sciences, University at Buffalo/SUNY, Buffalo, NY, United States; ^3^Department of Neurology and Rehabilitation Medicine, George Washington University, Washington, DC, United States

**Keywords:** ocular myasthenia gravis, myasthenia gravis diagnosis, myasthenia gravis treatment, OMG mimics, OMG

## Abstract

Ocular myasthenia gravis (OMG) has no agreed-upon diagnostic and management criteria, leading to misdiagnoses and often misguided treatments. The purpose of this minireview is to provide guidance to clinicians who encounter possible OMG patients. We cite relevant literature and the recommendations for diagnosis and therapy based on the authors’ extensive experience in OMG. This report provides sound recommendations based on the authors’ successes and failures, coupled with relevant literature in the myasthenia gravis field.

## Introduction

Myasthenia gravis (MG), an autoimmune neuromuscular disorder, affects approximately 20 per 100,000 individuals in the United States. While MG can affect any voluntary skeletal muscle, many patients present initially only with ptosis, diplopia, and/or eye closure weakness. Confirming the diagnosis of OMG can be challenging, as both ptosis and diplopia occur in other disease conditions. Tables 1, 2 provide guidance on diagnosing ocular myasthenia gravis (OMG) and differentiating it from other disorders that present with similar signs. When only ocular symptoms and signs are present, the OMG is used (MG Foundation of America Class I). Dividing OMG from generalized MG (GMG) may appear arbitrary, as they both fall along a continuum of MG, but unlike GMG, OMG does not pose a threat to life. However, OMG causes profound visual disability that compromises many aspects of life (1). New onset OMG is typically treated initially with pyridostigmine monotherapy, but corticosteroids are frequently added, as most patients do not achieve adequate symptom relief with anticholinesterase inhibition alone. Approximately 80% of OMG patients require years of treatment with corticosteroids or other immunosuppressant agents (2, 3). Long-term immunosuppression poses the risk of opportunistic infection and reduces vaccine efficacy. These and other drug-related adverse events require ongoing laboratory monitoring and cointerventions to prevent or treat complications (4–12).

**Table 1:** Ocular myasthenia gravis diagnostic evaluation.

Clinical manifestations	Ptosis in one or both upper lids that is not due to local lid disease and fatigues after 120 s or less. Extraocular muscle weakness (limitation) in one or both eyes not conforming to the innervation pattern of the third cranial nerve or restrictive myopathy. The eye muscle limitation typically, but not always, varies during the day or between exams. For any ocular muscle limitation of movement, typically, but not always, there is obvious fatigability (worsening with 120 s or less of gaze in the direction of the affected muscle). Extraocular motility limitation change should be two grades or more using a simple ordinal scale in 25% increments (where 0 is full movement, −1 is 25%, −2 is 50%, −3 is 75%, and −4 is 100% and does not pass midline limitation). Normal lower facial, neck, arm, and leg strength (orbicularis oculi weakness is allowed). No pupillary abnormality except for prior ocular disease or ocular surgery, or medications (e.g., pilocarpine and mydriatics).
Confirmatory tests	Abnormal serum AChR binding antibody level. Edrophonium test (13, 64), (in regions where it is available) and Prostigmin test (65) – improvement in extraocular motility by ≥2 grades in at least one muscle, or if no obvious ocular motility limitation, improvement in the prism cover test (PCT) tropia measurement in primary gaze. For PCT deviations of 10 prism diopters (PD) or greater, a 50% improvement (e.g., XT 20 reduced to 10 PD or less), and for a deviation <10 PD, an improvement of at least 4 PD. For PCT deviations of 10 PD or less, the injected agent must also resolve PD in primary and downward gaze. Note that the extraocular muscles are extremely sensitive to edrophonium. Small amounts of the drug are needed, and the typical full 10 mg trial could actually worsen the measurement and the diplopia (48). Abnormal repetitive nerve stimulation with a minimum decrement of 10% or single fiber EMG (SFEMG) with blocking of five pairs or increased jitter in any muscle (if other conditions that could generate these abnormalities have been excluded). Ptosis recovers with the ice test (applied 2 min to the ptotic lid) with > 2 mm improvement (47).

**Table 2:** Non-rare disorders that can mimic OMG.

Ptosis	Age-associated changes in eyelid (dermatochalasis, levator dehiscence). Contact lens-induced tarsal surface papillary conjunctival reaction. Palpebral fissure narrowing due to misdirection after old facial nerve palsy. Partial Horner’s syndrome. Lacrimal region mass or inflammation.
Diplopia or ocular motor limitation or imbalance	Decompensated strabismus, either from a known or unknown childhood disorder, due to the loss of compensatory mechanisms. Cranial nerve palsy, acute or chronic, or failure to recover due to a spread of comitance (46). Miller Fisher variant of Guillain–Barré syndrome. Thyroid eye disease. Internuclear ophthalmoplegia with or without a skew due to a brainstem lesion. Orbital inflammation. Orbital mass. Divergence insufficiency. Chronic external ophthalmoplegia Wernicke’s encephalopathy Oculopharyngeal muscular dystrophy

Furthermore, within a year, approximately half of all OMG patients develop GMG with weakness spreading to facial, bulbar, truncal, arm, and leg muscles (13–20). Unfortunately, recent literature has been influenced by a retrospective study reporting lower GMG conversion rates in a cohort evaluated at an unknown (likely late) time point, limited to patients with ocular dysfunction who did not require immunomodulatory therapy, and excluding individuals who developed GMG early (21). There are currently no treatments with class I evidence to prevent conversion from OMG to GMG, although retrospective analyses suggest that corticosteroids may be beneficial (see below) (22–25). Since the focus of this review is the diagnosis and management of OMG, we will not review the pathophysiology of MG (26), the available treatments for GMG, nor the explanation for eyelid and ocular muscle susceptibiity (27). We will not address the muscle disorders induced by immune checkpoint inhibitors, as these agents rarely cause OMG.

## Diagnosis of OMG

### Antibodies to postsynaptic antigens

Data from our OMG clinics show that 55% of the 164 OMG patients at presentation had elevated binding antibodies to the acetylcholine receptor (binding AChRAb). Most commercial radioimmunoassays for detecting binding AChR antibodies (AChRAb) have limited sensitivity, leading to variable reporting of seropositive frequency in OMG. Some patients who test seronegative on commercial assays are found to be seropositive when evaluated using a research-grade cell-based assay (28). Importantly, new-onset seropositive OMG patients, who are not taking immunomodulatory therapy, have a higher risk (OR, 6.33 [95% CI, 1.71–23.42]) of developing GMG during the first 2 years (13).

Isolated ptosis or extraocular muscle dysfunction is rarely associated with muscle-specific kinase antibodies (MuSKAb) (29). Notably, binding AChRAb levels do not correlate with the severity of OMG or GMG. OMG is associated with antibodies to lipoprotein-related protein 4 (LRP4Ab) or other neuromuscular junction proteins also appear to be quite rare (30, 31).

#### MG biomarker

There is no established biomarker for MG that predicts disease activity, response to therapy, or clinical outcomes (28, 32). While an abnormal AChRAb is diagnostic of MG in the appropriate clinical setting, serum levels do not correlate with disease severity or clinical outcome. MicroRNAs (miRNAs) regulate differentiation and activation of immune cells in innate and acquired immunity. Alterations in miRNA expression and function are associated with immune system dysregulation found in autoimmune diseases, including MG (33–43). In a prospective study on 96 OMG patients followed for 2 years, two miRNAs were higher in individuals who transitioned to GMG vs. those who remained purely ocular: miRNA-30e-5p (9.1 ± 0.5 vs. 6.3 ± 0.9; *p* < 0.0001) and miRNA-150-5p (7.4 ± 1.1 vs. 6.4 ± 1.1; *p* = 0.01) (43).

Recent data indicate that high levels of miRNA-30e-5p predict MG relapse (*p* = 0.049) in several MG subgroups, including patients with OMG at onset, with a hazard ratio of 2.81 (44). These data suggest miRNA assays may be useful biomarkers for predicting generalization and relapse.

#### OMG diagnostic criteria

Table 1 provides the diagnostic approach, including the potential for false positives and negatives, each of which can have consequences for the patient, given the visually disabling and potential for complications of therapy (2, 45). OMG patients should have at least one clinical manifestation of OMG and at least one confirmatory test for MG. Although.

OMG can affect only one eye; other disorders should be considered in the clinic (Table 2) (46).

Fluctuation in symptoms and signs of ptosis and ocular motility limitation is characteristic of OMG. We consider the icepack test part of the clinic assessment, given its sensitivity of 86% (47). If the criteria are not clearly satisfied or another disorder is suspected, additional testing may be required.

Confirmatory tests have limited investigation of their sensitivity and specificity (2, 48, 49), especially when performed by less-experienced individuals. This is particularly true for the ice pack test, which has 79% specificity. Edrophonium is no longer available in the United States, but each confirmatory test can be compromised if a minimal change in lid position or a subjective sensation of improvement is deemed a positive result. We would also caution that a pyridostigmine trial based only on subjective improvement is not a reliable diagnostic strategy.

Antibody testing also has limitations. Binding AChRAbs are found in approximately 85% of generalized and 50% of OMG cases. Modulating and blocking AChRAbs adds no diagnostic value and can be false positives. Minimally elevated binding AChRAbs levels can also be a false positive (50). MuSKAbs occur in approximately one-third of AChRAb-negative GMG patients and rarely in OMG. LRP4 antibodies occur in 2–5% of otherwise seronegative GMG patients but lack specificity, as they may also appear in motor neuron disease. Electrophysiologic studies help confirm impaired neuromuscular transmission, particularly in seronegative or clinically unclear cases. Repetitive nerve stimulation shows a decremental response in approximately 75% of patients. Single-fiber EMG has >95% sensitivity but can yield false positives due to a variety of neuropathic and myopathic conditions, prior botulinum toxin treatment, and borderline abnormalities even in normal individuals.

Laboratory, ice application, and electrophysiologic testing should be guided by a clinical syndrome compatible with OMG to avoid misdiagnosis and the wrong treatment.

### Clinic evaluation

Patients should be evaluated at each clinic visit using the following assessments:

1. Questions probing and examination to detect the development of GMG.
2. Determine if the ptosis improved enough to unblock the visual axis.
3. Ocular motor assessment.

- Approximate amount of time per day diplopia interferes with watching television, reading, computer work, and driving.
- Examination of ocular versions and ductions and determination if there is diplopia in primary, right, and left gaze and downgaze at distance and at near for reading. If possible, prism cover test measurements can provide objective measurements correlated with diplopia.

## Treatment of OMG

The primary treatment strategy for OMG is to minimize or eliminate ptosis that interferes with vision and to improve or fully restore binocular visual function to allow for normal activities of daily living. A secondary concern is considering interventions that may reduce the risk of developing GMG. There are no prospective studies that have established effective treatments to prevent the transition from OMG to GMG. It should be noted that durable remissions with or without pharmacological therapy occur in up to 22% of GMG (18) but less than 5% of OMG adult patients (MJK unpublished data). As OMG is not in itself life-threatening, major adverse effects or complications of therapies must be avoided. The approach is to reduce medications to the lowest doses needed to maintain an adequate clinical response, even if mild symptoms such as diplopia on far lateral gaze remain.

Retrospective data suggest that prednisone delays or reduces conversion to GMG (13, 22, 25, 51), as may other immunosuppressant drugs (52). We cannot extrapolate using therapies shown to be effective for GMG to the treatment of OMG, as prior GMG treatment trials have almost exclusively been conducted on binding AChRAb-positive participants. However, case series suggest that seronegative GMG patients benefit from thymectomy (53) or intravenous immunoglobulin therapy (54) with similar frequency to those who are seropositive.

Mechanical and optical remedies for OMG are underutilized, but most are not durable, given the fluctuation in the disease. These include eyelid or extraocular muscle surgery options. These have a role in patients judged to be refractory to pharmacological approaches, at times due to irreparable postsynaptic injury. Eyelid surgery to unblock the visual axis must be tempered with the risk of poor eyelid closure and secondary breakdown of the cornea. Eye muscle surgery should only be considered when the ocular movement limitations and measured separation are unchanged over months. Lid crutches attached to eyeglass frames can help, but commonly irritate the eyelids and lead to cornea/conjunctiva drying and exposure. Paste-on prisms can alleviate diplopia and can be changed over time to reflect shifts in ocular misalignment.

However, Fresnel prisms are not optically clear, and some patients do not tolerate them. Furthermore, ocular misalignment can be both horizontal and vertical or vary depending on the direction of gaze, limiting the usefulness of a Fresnel prism. Prisms ground into the glasses are optically clear but are not useful if the ocular separation fluctuates or varies with the gaze direction.

Below, we briefly discuss pharmacological and surgical interventions for OMG, for which there is considerable experience.

### Pyridostigmine

Pyridostigmine facilitates neuromuscular transmission by inhibiting the catalysis by acetylcholinesterase, which increases the amount of acetylcholine at the neuromuscular junction. Pyridostigmine typically improves ptosis more than diplopia. Unfortunately, in cases where OMG is initially controlled by pyridostigmine, symptoms recur in the majority of weeks to months. Only approximately 3% of OMG patients, typically older and male, experience pharmacological control lasting years with pyridostigmine alone.

### Prednisone

Prednisone, an oral corticosteroid, remains a mainstay of management in both OMG and GMG due to its effectiveness, ease of administration, and low cost (17, 24).

We *caution against immediately starting high-dose oral or intravenous corticosteroids due to the risk of disease exacerbation that can evolve to a myasthenic exacerbation with generalization.* The prednisone regimens for OMG described below are based on prospective open-label studies (2, 13) and incorporate feedback from both neuroophthalmologists and neuromuscular neurologists.

Our prospective observational data demonstrate that prednisone restores binocular motor function in as little as 1 month (22). However, to maintain the benefit, most OMG patients require prednisone for many months, typically at an average of 6 mg daily over 2 years (2). In our recent study that followed 105 OMG patients (who did not develop GMG in the first 2 years) for a mean of 8.2 ± 4.9 years, only 21% did not require prednisone or other forms of immunosuppression long-term. Approximately 40% required a steroid-sparing immunosuppressant even in the absence of GMG (6). Daily prednisone requirements at 1 year were similar for the 45 seropositive and 45 seronegative OMG patients (3.75 mg ± 4.45 vs. 2.58 mg ± 2.95, respectively; *p* = 0.33).

Experts use a variety of prednisone protocols, and we suggest three potential treatment courses. The first regimen has not been associated with any paradoxical MG worsening. Patients start oral prednisone 10 mg daily for 2 days, increase to 20 mg daily for the next 2 days, then transition to 40 mg daily for 1 week, followed by a taper of 5 mg per week until reaching 20 mg. Thereafter, the dose is reduced by 2.5 mg weekly to 10 mg daily. If OMG remains adequately controlled as confirmed on a clinic visit, further tapering ensues using 2.5 mg or 1 mg decremental steps. The second approach uses alternate-day dosing for titrating to a 40-mg dose of prednisone on alternate days for 2 to 4 weeks, followed by a taper that follows a similar schedule described earlier. The third regimen starts with prednisone 10 mg daily for 3 days, with a 5 mg increase every 3 days until 25 mg daily is reached. If there is a dramatic improvement at any dose, the dose is held at that dose for 1 month. Otherwise, the dose is escalated to 40 mg in 5 mg increments weekly. Once symptoms are controlled, the dose is tapered to 5 mg per month. Drug reduction is faster if the patient experiences significant side effects. At 10 mg, the taper is slowed to 2.5 mg weekly until 5 mg, where the dose is held long-term.

Of note, if doses higher than 40 mg (the maximum used is 50 mg) are used, the dose is tapered to 40 mg daily, followed by the dose reduction schedule above. *Below, we summarize strategies for handling disease fluctuations.*

1. Minor flare: OMG symptoms may return when prednisone is tapered below 10 mg daily. Corresponding exam findings include ptosis obscuring vision. We allow for diplopia in gaze directions that do not interfere with normal function. The treatment goal is to restore and maintain vision function in primary and downward gazes, the directions of gaze most associated with satisfactory quality of life. For minor flares, we suggest an increase in prednisone by 10 mg a day for 1–2 weeks. Symptoms often resolve with this dose escalation, and the tapering schedule above is resumed.
2. Disease recurrence: If symptoms and signs relapse during a second tapering attempt (e.g., after following the protocol above for a minor flare) or after prednisone has been tapered off entirely, our approach resumes prednisone at 20 mg daily for 2 weeks; if disease control is restored, a taper of 2.5 mg every week to 10 mg daily is employed. The patient is assessed after 4 weeks on the 10 mg daily dose, with tapering resumed in steps of 2.5 to 1 mg a week if there are no symptoms. If disease control does not follow the dose escalation to 20 mg daily, the initial treatment protocol with dosing up to 40 mg a day can be used, followed by the taper schedule mentioned earlier.
3. We define prednisone failures as follows: (1) If there is inadequate OMG control, (2) either due to lack of improvement or maintenance of binocular visual function when the prednisone daily dose is 10 mg or less, and 3. if patients develop GMG.

Additional therapies for OMG patients who cannot tolerate or fail to take prednisone are listed below.

### Azathioprine

Azathioprine is commonly used as a non-corticosteroid immunosuppressive agent for OMG patients who fail prednisone, experience intolerable or unmanageable adverse events, or cannot take corticosteroids (e.g., type 1 diabetes mellitus) (3). Serum thiopurine methyltransferase activity should be measured to avoid potential irreversible hematopoietic complications. There are no prospective clinical trials of azathioprine in OMG. Most patients eventually require doses of 2–3 mg/kg daily (200 to 250 mg daily are often needed), which can be attained after 4 to 8 weeks if the patient tolerates lower doses beginning with 50–100 mg daily. Favorable clinical impact from azathioprine may take 6 months or longer. Laboratory monitoring with a complete hemogram and liver and renal function tests is required. Monitoring begins on a schedule of every other week, and when stable, every 6 months for chronic therapy. Case reports suggest that azathioprine can increase the risk of hematological and skin cancers, but larger studies show either no or a small risk (54–56). Interestingly, there are no medical reviews about the adverse effects of azathioprine on the Drugs@FDA-approved drug site. Many, but not all, OMG patients will continue taking low-dose prednisone (typically less than 7.5 mg daily) while on azathioprine.

### Mycophenolate mofetil

Mycophenolate mofetil is also commonly used as a non-corticosteroid immunosuppressive agent. Two authors (HK and GW) prefer this agent for individuals who fail prednisone. There are no prospective studies or large case series on mycophenolate use in OMG, with experience limited to case reports. Most OMG patients require a dose of 2000–2,500 mg daily.

Improvement in OMG can occur in several months, but, like GMG, the benefit may not be seen for at least a year (57). As with azathioprine, many but not all patients will continue on low-dose prednisone. Laboratory monitoring with a complete hemogram is required every 2 to 4 weeks for the first 2 months and then every 6 months.

### Tacrolimus

Tacrolimus is used in similar situations to azathioprine and mycophenolate mofetil. Case series, mostly from Japan and China, demonstrate that tacrolimus improves OMG control in children, particularly those resistant to corticosteroid therapy (58, 59). It is primarily used in GMG, but it is an option for OMG. Laboratory monitoring is similar to the schedule for azathioprine. Hyperglycemia is also a potential adverse event.

### Intravenous immunoglobulin (IVIG) and plasma exchange

Although IVIG and plasma exchange are used in the management of myasthenic crisis and in the chronic management of GMG, evidence for a favorable impact on OMG is extremely limited. There are no prospective studies or reliable case series to support the use of IVIG or plasma exchange in OMG. Given the expense, potential safety issues, and lack of clinical evidence, we do not recommend either in the management of OMG.

### Thymectomy

The prospective, randomized, multinational trial proved that transsternal thymectomy is effective in improving muscle function and reducing corticosteroid requirements in GMG (60, 61). Our group proposed a trial to assess the impact of minimally invasive thymectomy early in the disease course of OMG, with the hypothesis that it would reduce corticosteroid requirements, potentially induce remission in early disease states, and reduce the risk of deterioration in GMG. At present, the experience of thymectomy in OMG is limited to small observational studies, and often it is performed late in the disease course. One large retrospective study reported the benefit of thymectomy in an OMG population with a profoundly high frequency of thymoma (62). We do not consider thymectomy as the standard of care in non-thymomatous OMG cases.

### Targeted therapies currently approved for only GMG

Several agents that inhibit either the complement cascade or neonatal Fc receptor recycling of IgG have been approved in GMG (63). Studies are planned or in progress to assess the impact of some of these agents in patients with OMG or who have GMG with prominent OMG problems. Based on ocular subscores performed during the pivotal GMG clinical trials and corticosteroid-sparing effects seen during open-label extensions, we surmise that these agents should be effective in managing OMG. Given the high cost, requirement for needle-based delivery, and mandatory vaccination for *Neisseria meningitides* if a complement inhibitor is used, we would expect that the main use of these new therapies will be for more refractory OMG cases.

## Conclusion

The diagnosis of OMG remains challenging, as diplopia and ptosis are encountered in a variety of neurological, ophthalmological, and medical disorders. Pharmacological approaches to managing OMG are largely based on expert opinions and literature that has focused on GMG. In this brief review, we summarized our approach to diagnosing and managing OMG. For management to evolve further with regulatory approval for specific agents in OMG, this group of patients needs to be included in therapeutic trials that include both binding AChRAb seropositive and seronegative patients.
